# Mechanism for *de novo* initiation at two sites in the respiratory syncytial virus promoter

**DOI:** 10.1093/nar/gky480

**Published:** 2018-06-05

**Authors:** Tessa N Cressey, Sarah L Noton, Kartikeya Nagendra, Molly R Braun, Rachel Fearns

**Affiliations:** Department of Microbiology, Boston University School of Medicine, Boston, MA 02118, USA

## Abstract

The respiratory syncytial virus (RSV) RNA dependent RNA polymerase (RdRp) initiates two RNA synthesis processes from the viral promoter: genome replication from position 1U and mRNA transcription from position 3C. Here, we examined the mechanism by which a single promoter can direct initiation from two sites. We show that initiation at 1U and 3C occurred independently of each other, and that the same RdRp was capable of precisely selecting the two sites. The RdRp preferred to initiate at 3C, but initiation site selection could be modulated by the relative concentrations of ATP versus GTP. Analysis of template mutations indicated that the RdRp could bind ATP and CTP, or GTP, independently of template nucleotides. The data suggest a model in which innate affinity of the RdRp for particular NTPs, coupled with a repeating element within the promoter, allows precise initiation of replication at 1U or transcription at 3C.

## INTRODUCTION

An intriguing facet of the non-segmented negative strand RNA viruses (nsNSVs) is that their RNA dependent RNA polymerase (RdRp) is able to use the viral genome RNA as a template for two different RNA synthesis processes: transcription, which yields subgenomic capped and polyadenylated mRNAs and genome replication (1,2). In the case of respiratory syncytial virus (RSV), a major cause of human respiratory disease (3–6), transcription and replication both begin at the *leader* (*le*) promoter region at the 3′ end of the viral genome (7–9), but they are initiated at different sites within the *le* (1,10). Both processes are dependent on a core promoter element located within the first 11 nt of the *le* (11). To transcribe the genome, the RdRp initiates RNA synthesis at position 3C of the template (T3C). After a short distance (∼25 nt), the RdRp releases the nascent RNA, but remains attached to the template, and scans to the *gene start* (*gs*) signal for the first gene (1,10,12). Here the RdRp reinitiates RNA synthesis and transcribes the remainder of the genome by stopping and restarting RNA synthesis at the gene junctions (13,14). To replicate the genome, the RdRp initiates RNA synthesis opposite the 3′ terminal nucleotide of the *le*, at position T1U. Unlike the RNA initiated at T3C, RNA initiated at T1U becomes encapsidated with the viral nucleoprotein (N) (10). There is evidence to suggest that encapsidation is aided by the presence of 5′ AC, which is present at the end of the RNA initiated at T1U, but not the RNA initiated at T3C (15). Concurrent encapsidation causes the RdRp to become super-processive, allowing it to synthesize RNA continuously to the end of the genome (16,17). The *trailer* (*tr*) promoter at the 3′ end of the antigenome is identical in sequence to the *le* for 11 of the first 13 nt (positions 4 and 12 differ), and also signals initiation from T1U and T3C to produce full-length genome RNA and a ∼25 nt RNA, respectively (18–20). The small ∼25 nt trailer-specific transcript might function to subvert cellular stress granule responses (21).

While these findings explain how RSV replication and transcription are initiated, they raise the question, how does the RdRp initiate from two different sites within the same promoter? The RSV RdRp consists of a complex of two proteins, the large polymerase subunit (L), which contains the enzymatic domains for synthesizing, capping and methylating the mRNA, and phosphoprotein (P) (19,22,23). Studies using purified RSV RdRp in an *in vitro* RNA synthesis assay showed that the L-P complex alone can initiate at the T1U and T3C sites (10,19), indicating that other RSV proteins required specifically for transcription or replication, M2-1 and N (16,24,25), do not determine initiation site selection. However, it is conceivable that there are distinct sub-populations of L-P that allow initiation either at T1U or T3C. Alternatively, the same RdRp might be capable of initiating at either site; if so, there must be a factor that determines the relative frequencies with which the two initiation sites are used. Studies using a cell-based minigenome system, in which RNA replication was limited to a single step, showed that if a pyrimidine substitution or a deletion was introduced at position 1 of the *tr* promoter, the RdRp could still produce replication product at ∼60% of wt levels. Remarkably, almost all the replication products were initiated at the T1 position relative to the wt promoter sequence, with the wt nucleotide, ATP (26). Similar results were obtained with the *le* promoter (15). In addition, in a study in which the first nucleotide of an internal *gs* signal was substituted, the mRNA products were initiated with wt GTP with relatively high frequency (27). These studies suggest that the RSV RdRp has an affinity for ATP or GTP, independent of the template nucleotides. If this were the case, this could be an important factor in allowing the RdRp to select the +1 and +3 initiation sites with the appropriate frequencies. However, a caveat to these minigenome experiments is that because RNAs analyzed were isolated from cells, the analysis would have been biased toward detection of stable RNAs. As the sequence at the 5′ ends of the replication and transcription products would be expected to affect encapsidation and capping efficiency, respectively, and thus RNA stability, a relatively low level incorporation of non-templated ATP or GTP could have been magnified. In this study, we utilized an *in vitro* assay to examine the mechanism of initiation at the RSV promoter. In this system, results would not be influenced by the relative stabilities of the RNA products. This analysis revealed that the same RSV RdRp is able to initiate at +1 or +3. The data also show that that the RdRp has an innate affinity for ATP and CTP, or GTP, and that association with NTPs guides initiation site selection. These findings explain how the RSV RdRp is able to initiate transcription and replication from a single promoter.

## MATERIALS AND METHODS

### Primer extension analysis of RSV specific RNAs from infected cells

HEp-2 cells were infected with RSV A2 at an MOI of 3 or mock infected and incubated at 37°C for varying times. Total intracellular RNA was isolated using Trizol (Invitrogen), as described previously (10). Primer extension reactions were performed using primers that hybridized to nucleotides 15–39 or 13–35 relative to the 5′ termini of the antigenome or genome RNAs, respectively, as described previously (10,15). Primer extension products were compared to ^32^P-end labeled DNA oligonucleotides of sequence and length equivalent to cDNAs corresponding to RNAs initiated at T1U and T3C.

#### Purification of the RSV L-P complex

**Figure 1.**
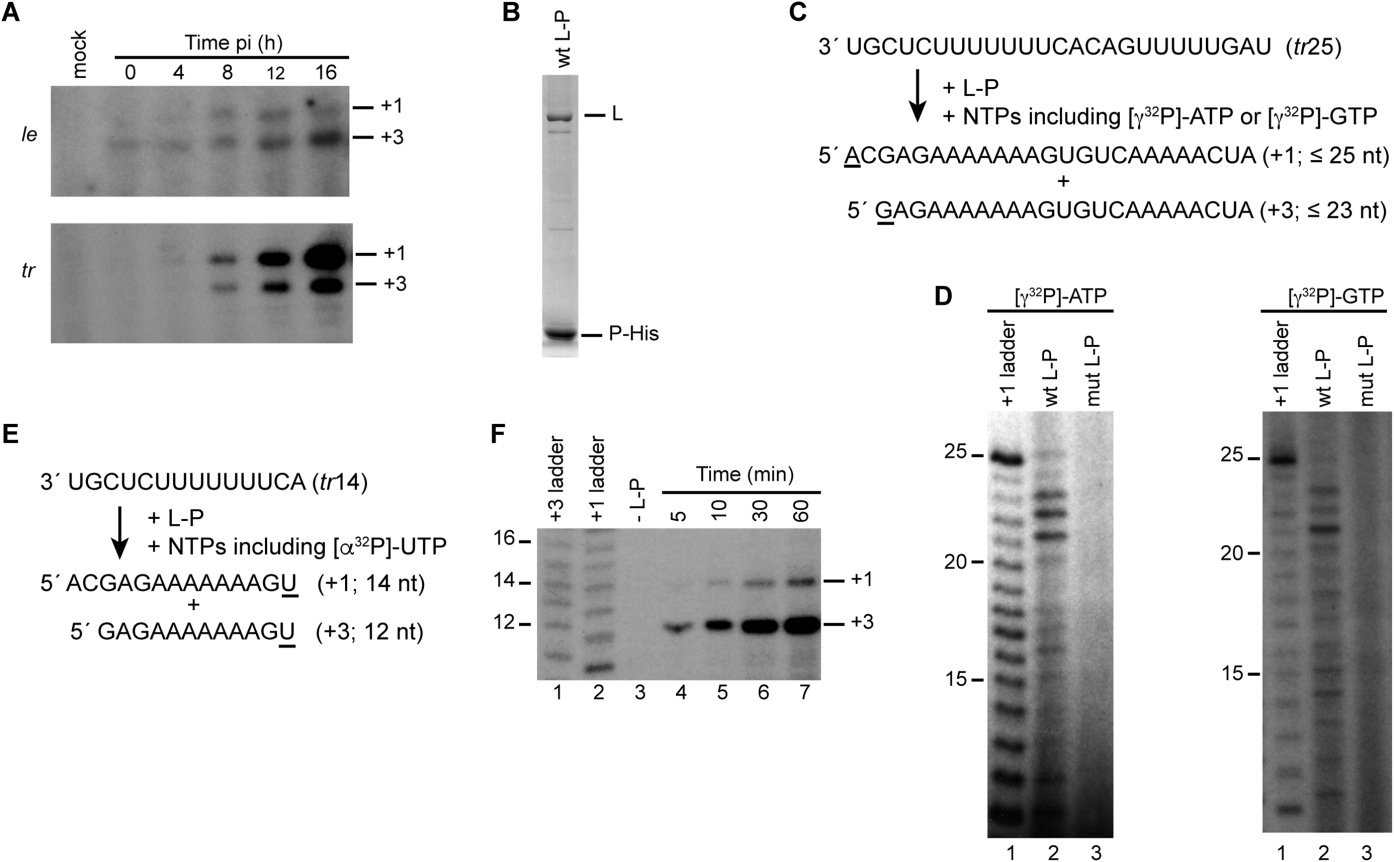
The RSV polymerase initiates precisely at T1U and T3C. (**A**) Primer extension analysis of RSV RNAs produced from the *le* and *tr* promoters, isolated from infected cells at different times post infection, as indicated. Bands representing RNAs initiated at the T1U and T3C initiation sites (indicated with +1 and +3, respectively) were identified by comparison to ^32^P-end labeled DNA oligonucleotides of equivalent sequence and length (10,12,26). (**B**) SDS-PAGE of purified recombinant wt L-P complexes stained with colloidal blue stain. (**C**) Schematic diagram illustrating how RNAs initiated with ATP or GTP were identified. The template consisted of the first 25 nt of the *tr* promoter (*tr*25); sites of radiolabel incorporation in the RNA products are underlined. (**D**) RNA products generated in the presence of [γ^32^P]-ATP or [γ^32^P]-GTP (left and right panels, respectively). Reactions contained 500 μM ATP, 1 mM CTP, 10 μM GTP and 500 μM UTP (left panel) or 500 μM each NTP (right panel) and were incubated for 3 h. In each case, lane 1 is a ladder consisting of a 25 nt [γ-^32^P] labeled RNA corresponding in sequence to RNA initiated at the +1 site, which had been subjected to alkali digest, and lane 3 is a negative control reaction performed with catalytically inactive D811A L protein. (**E**) Schematic diagram illustrating how RNAs initiated at T1U or T3C were identified. The template consisted of the first 14 nt of the *tr* promoter (*tr*14), sites of radiolabel incorporation in the RNA products are underlined. (**F**) RNA products generated in the presence of [α^32^P]-UTP. Reactions contained 1 mM each of ATP, CTP and GTP, and [α^32^P]-UTP tracer. Lanes 1 and 2 show ladders consisting of 23 or 25 nt [γ-^32^P] labeled RNA corresponding in sequence to RNA initiated at the +3 or +1 sites, respectively, which had been subjected to alkali digest. Lane 3 is a negative control reaction in which the L-P complex was omitted from the reaction.

A codon-optimized version of the RSV (strain A2) L protein ORF was expressed in insect cells as previously described (19). L-P protein complexes were isolated from cell lysates by affinity chromatography on Ni-NTA agarose resin (ThermoFisher). The resin was washed three times with 60 mM imidazole, two times with 100 mM imidazole, and the L-P complex was either eluted using TEV protease, or with 250 mM imidazole. The L-P preparation was then subjected to dialysis. Isolated L-P complexes were analyzed by SDS-PAGE and PageBlue staining (Fermentas) and the L protein concentration was estimated against bovine serum albumin reference standards. Experiments shown in Figures 1, 4, 5 and Supplementary Figure S1 were performed with both types of L-P preparations (no difference in experimental results between preparations was observed). Remaining experiments were performed with the TEV eluted L-P preparations.

#### *In vitro* RNA synthesis reactions to detect transcripts initiated at T1 and T3

Reactions performed with *tr*25 templates and [γ^32^P]-ATP tracer were performed in 50 μl reactions with the following conditions: 2 μM RNA templates (Dharmacon), 50 mM Tris pH 7.4; 8 mM MgCl_2_; 5 mM DTT; 10% glycerol, 500 μM ATP, 1 mM CTP, 500 μM UTP and 10 μM GTP with 10 μCi [γ^32^P]-ATP (6000 Ci/mmol). Reaction mixes were heated to 30°C for 5 min prior to addition of L-P complexes containing 100–300 ng of L protein. Reactions were incubated for 3 h at 30°C then incubated at 90°C for 3 min to inactivate the polymerase. Following heat inactivation, samples were incubated at 30°C with 1 μl of 5′ monophosphate dependent exonuclease for 1 h to remove RNA that had become radiolabeled by a contaminating kinase activity. Reactions performed with [γ^32^P]-GTP tracer were performed under similar conditions, except that they contained 500 μM each NTP and 10 μCi [γ^32^P]-GTP (6000 Ci/mmol).

Reactions performed with *tr*14 templates and [α^32^P]-UTP tracer were performed in 50 μl reactions with the following conditions: 2 μM RNA templates (Dharmacon), 50 mM Tris pH 7.4; 8 mM MgCl_2_; 5 mM DTT; 10% glycerol, 1 mM each ATP, CTP and GTP (unless stated otherwise) and 10 μCi [α^32^P]-UTP (3000 Ci/mmol). Reaction mixes were heated to 30°C for 5 min prior to addition of L-P complexes containing 100–300 ng L protein. RNA synthesis reactions were incubated at 30°C for 30 min (unless indicated otherwise) then incubated at 90°C for 3 min to inactivate the polymerase. Reactions were briefly cooled on ice and combined with 10 U of calf intestinal phosphatase (NEB) and incubated at 37°C for 1 h to remove 5′ terminal phosphates and reduce the background signal from unincorporated [α^32^P]-UTP.

For all reactions, RNA was extracted with phenol-chloroform and ethanol precipitated. Pellets were resuspended in RNase free water, and an equal volume of stop buffer (deionized formamide containing 20 mM EDTA, bromophenol blue, xylene cyanol) was added. Molecular weight ladders representing products initiated from +1 or +3 sites were prepared as described previously (19). RNA samples were analyzed by electrophoresis on a 20% polyacrylamide gel containing 7 M urea in Tris-borate-EDTA buffer, followed by autoradiography. Data were quantified by analyzing TIF images of the autoradiograms using Quantity One software (Biorad) as described previously (4), except for that shown in Figure 3, in which the data were collected by phosphorimage analysis. In some of the images presented, the contrast was adjusted using the brightness/ contrast function in Adobe Photoshop. Any adjustments made were applied to the entire image.

### *In vitro* RNA synthesis reactions to detect pppAC primer formation

Reaction mixes contained 2 μM RNA oligonucleotide (Dharmacon); 50 mM Tris, pH 7.4; 8 mM MnCl_2_ or MgCl_2_; 5 mM DTT; 10% glycerol; 500 μM (each) ATP and CTP and 100 μM (each) GTP and UTP with 2 μl [α-^32^P]-CTP in a final volume of 50 μl. Reaction mixtures were preincubated prior to addition of L-P protein as described above, incubated at 30°C for 3 h and then heat inactivated as described above. Reactions were combined with an equal volume of deionized formamide containing 20 mM EDTA, bromophenol blue, and xylene cyanol, and analyzed by electrophoresis on a 25% polyacrylamide gel containing 6 M urea in Tris–taurine–EDTA buffer.

## RESULTS

### The L-P complex and a 14 nt promoter sequence are sufficient for precise initiation at T1U and T3C

Primer extension analysis of RNA isolated from RSV-infected cells showed that T1U and T3C were the only initiation sites detected in both the *le* and *tr* promoters, suggesting that initiation site selection is precisely controlled (Figure 1A). To characterize the mechanism underlying this control, RSV RdRp activity was reconstituted *in vitro* with an RNA oligonucleotide template. This assay recapitulates events that occur during infection, after N is displaced from the promoter (12,19,28). Purified L-P complexes (Figure 1B) were incubated with an oligonucleotide containing nucleotides 1–25 of the *tr* promoter, and NTPs, including either [γ^32^P]-ATP or [γ^32^P]-GTP. Incorporation of [γ^32^P]-ATP or [γ^32^P]-GTP at the 5′ position of the RNA product would result in radiolabeled RNA transcripts (Figure 1C). Both [γ^32^P]-ATP containing RNAs up to 25 nt in length, and [γ^32^P]-GTP containing RNAs up to 23 nt in length were produced (Figure 1D), consistent with initiation from T1U and T3C, respectively (small amounts of longer [γ^32^P]-GTP products could be due to stuttering of the RdRp on the U tracts within the template, as described previously (4)). The products smaller than 25 and 23 nt could be RNA transcripts that were initiated at T1U and T3C, but that were not elongated to the end of the template. However, they could be RNAs initiated from other U or C residues within the promoter. Thus, this experiment did not show if the L-P complex alone was sufficient for precise initiation, meaning initiation only at positions T1U and T3C. Previous studies had shown that there was little difference in RNA synthesis activity from *tr* templates ranging from 12–25 nt in length (4). Therefore, to determine if L-P was sufficient for precise initiation we used a shorter *tr*14 template, in which the first A residue was at the 5′ end, and [α-^32^P]-UTP as the tracer nucleotide. In this case, radiolabel would only be incorporated into the RNA products when the RdRp reached the end of the template (Figure 1E). Products of 14 and 12 nt were detected, indicating that the RdRp initiated precisely at T1U and T3C, and not at other sites within the promoter (Figure 1F). This result indicates that all the bands labeled with [γ^32^P]-ATP and [γ^32^P]-GTP in Figure 1D represent RNA initiated at T1U and T3C, respectively. The experiment presented in Figure 1F showed that the 12 nt product was more abundant than the 14 nt product, indicating that the RdRp preferred to initiate at T3C. The ratio of products initiated at T1U and T3C *in vitro* was different from what was observed by primer extension analysis of RNA from the *tr* promoter in RSV infected cells. The difference between the relative amounts of +1 and +3 products in the *in vitro* assay compared to RSV infected cells could be due to the relative stabilities of the RNAs in the cellular environment, in which the encapsidated +1 replication product would be expected to be very stable, or could be due to technical reasons; for example, because the RNA initiated at T3C is only extended ∼25 nt, it might not bind as efficiently to the primer used for primer extension as the RNA initiated at T1U.

Attempts were also made to examine initiation from the *le* promoter using the same approach as illustrated in Figure 1E, with [α-^32^P]-UTP used as the tracer nucleotide. However, because of a significant bias towards initiation at T3C compared to T1U, and because the minimal *le* promoter required for detectable initiation from T1U contained the first UTP incorporation site before the end of the template, it was technically less tractable to study than the *tr* promoter. Nonetheless, the analysis showed that the *le* promoter also signaled initiation from T1U and T3C, similarly to the *tr* promoter (Supplementary Figure S1).

### Initiation site selection was governed by ATP versus GTP concentration

**Figure 2.**
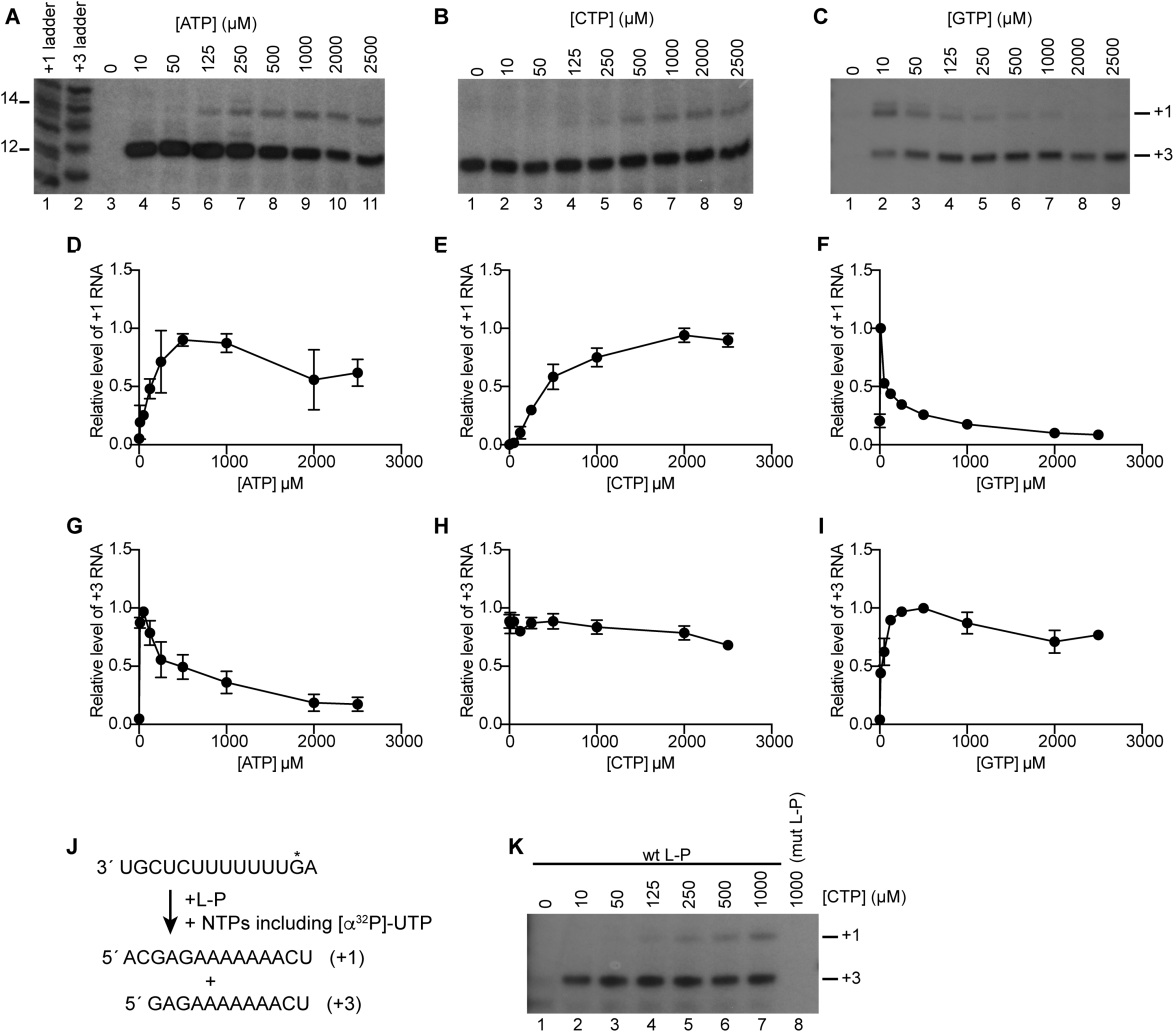
NTP requirements for initiation at T1U versus T3C. (**A**–**C**) RNA synthesis reactions were performed using the scheme shown in Figure 1E. The reactions contained 1 mM each of ATP, CTP and GTP, except for the NTP being titrated, which was included at the indicated concentration; the [α^32^P]-UTP trace was at 60 nM. Lanes 1 and 2 of panel A contain ladders as described for Figure 1. (**D**–**I**) Quantification of the products from the +1 (D–F) and +3 (G–I) initiation sites. The data were normalized to the maximum level of product from the +1 and +3 initiation sites in each titration, and the graphs show the mean and standard error of three (D, I) or four (E, F, G, H) independent experiments. (**J**) Schematic diagram illustrating the experimental design for examining the effect of CTP on elongation versus initiation. RNA synthesis reactions were performed using a *tr*14 template with a position 13 C-to-G substitution (indicated with an asterisk), such that products from both the +1 and +3 initiation sites would require CTP to be elongated to the end of the template. (**K**) RNA synthesis reactions were performed on the template shown in J, with conditions described for A–C. Lane 8 is a negative control in which the L-P complex contained a D811A substitution in the L protein. This panel is representative of two independent experiments.

Given that initiation at T1U and T3C requires ATP and GTP as the initiating NTP, respectively, we examined how NTP concentration affected the relative levels of RNA produced from the two sites. For this, we used the *tr*14 template, and measured relative levels of 14 and 12 nt products as surrogates for relative levels of initiation from +1 and +3. While a caveat to this approach is that these are products of both initiation and elongation steps of RNA synthesis, we rationalized that elongation of the RNAs initiated at T1U and T3C would be similarly affected by variations in NTP concentration so that differences observed would be due to effects on initiation. The concentrations of ATP, CTP or GTP were individually varied from low to high concentration, while maintaining the other two NTPs at high concentration (Figure 2A–C). Initiation at T3C was dominant compared to initiation at T1U, under all NTP concentrations tested, except those in which the GTP concentration was very low (Figure 2C, lane 2), demonstrating that the RdRp had a strong preference for initiating at T3C.

The data obtained provide information on the relative requirements for different NTPs for the two initiation events under these assay conditions. For optimal initiation at T3C, the initiating NTP, GTP, was required at a relatively high concentration (250–500 μM), whereas the second NTP to be incorporated (NTP 2), ATP, was required at a significantly lower concentration of 50 μM (Figure 2I and G; Supplementary Figure S2, F and D). For optimal replication initiation at T1U, ATP was required at 500 μM, but NTP 2, CTP was required at a very high concentration (2 mM; Figure 2D and E; Supplementary Figure S2, A and B). Other studies have shown that the *K*_m_ for CTP during elongation is lower than for other NTPs (29) and a control experiment, with a template containing an inserted G residue near the 5′ end, confirmed that a high concentration of CTP was required specifically for initiation, and not elongation (Figure 2J, K). This indicates that recruitment of CTP presented a barrier to initiation at T1U. In contrast, CTP concentration had no effect on initiation from +3 demonstrating that initiation at +3 did not depend on prior initiation at T1U (Figure 2B, H).

**Figure 3.**
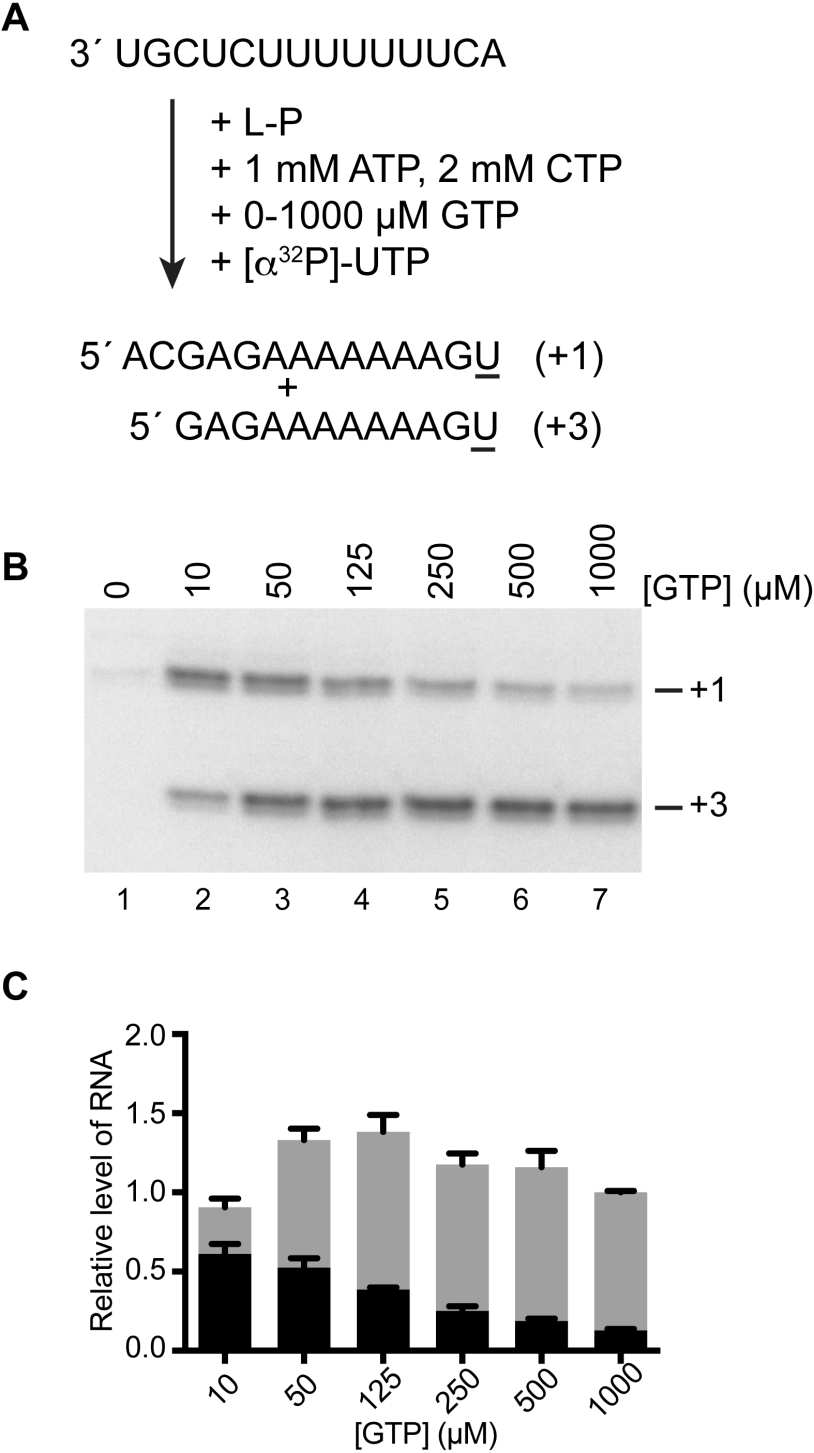
There is an inverse relationship between initiation at T1U or T3C, dependent on NTP concentration. (**A**) Schematic diagram illustrating the experimental design. The reactions contained 1 mM ATP, 2 mM CTP and the GTP concentration was varied from 0 to 1000 μM; the [α^32^P]-UTP trace was at 60 nM. (**B**) RNA synthesis products from the reactions. (**C**) Stacked bar chart showing quantification of the products initiated from the +1 and +3 sites. The levels of RNA initiated at +1 or +3 are shown in black and grey, respectively. The data obtained from replicate experiments were normalized such that the total level of RNA at 1000 μM GTP was 1. The data show the mean and standard error of three independent experiments.

In addition to providing information regarding NTP requirements for initiation, the data showed that variation of NTP concentration affected initiation site selection: increasing ATP caused an increase in RNA initiated at +1 and a decrease in RNA initiated at +3 (Figure 2A, D, G; Supplementary Figure S2 A and D) whereas increasing GTP caused the opposite effect (Figure 2C, F, I; Supplementary Figure S2, C and F). Given that the template is in significant molar excess compared to the RdRp, this result suggests that the same population of RdRp was capable of initiating at the two sites, and that initiation site selection was determined by binding either ATP or GTP. However, even under low GTP concentrations that were the most favorable for initiation from +1 versus +3, the level of initiation from +1 was still relatively low (Figure 2C, lanes 2 and 3). It was possible that the reason for this was that 1 mM CTP was insufficient to support efficient initiation from +1. To examine this possibility, GTP concentration was varied from 10 to 1000 μM, in the presence of 1 mM ATP and 2 mM CTP (Figure 3A). Under these conditions, initiation at +1 was dominant at low concentrations of GTP (Figure 3B). Quantification of the levels of RNA initiated at +1 and +3 showed that the total level of RNA product was similar at all GTP concentrations tested, and that there was an inverse correlation between the levels of RNA initiated at +1 versus +3 (Figure 3C). This result demonstrates that the RdRp could be switched between initiation sites by either ATP or GTP binding.

### The RSV RdRp was constrained to initiate opposite a C or UG motif

**Figure 4.**
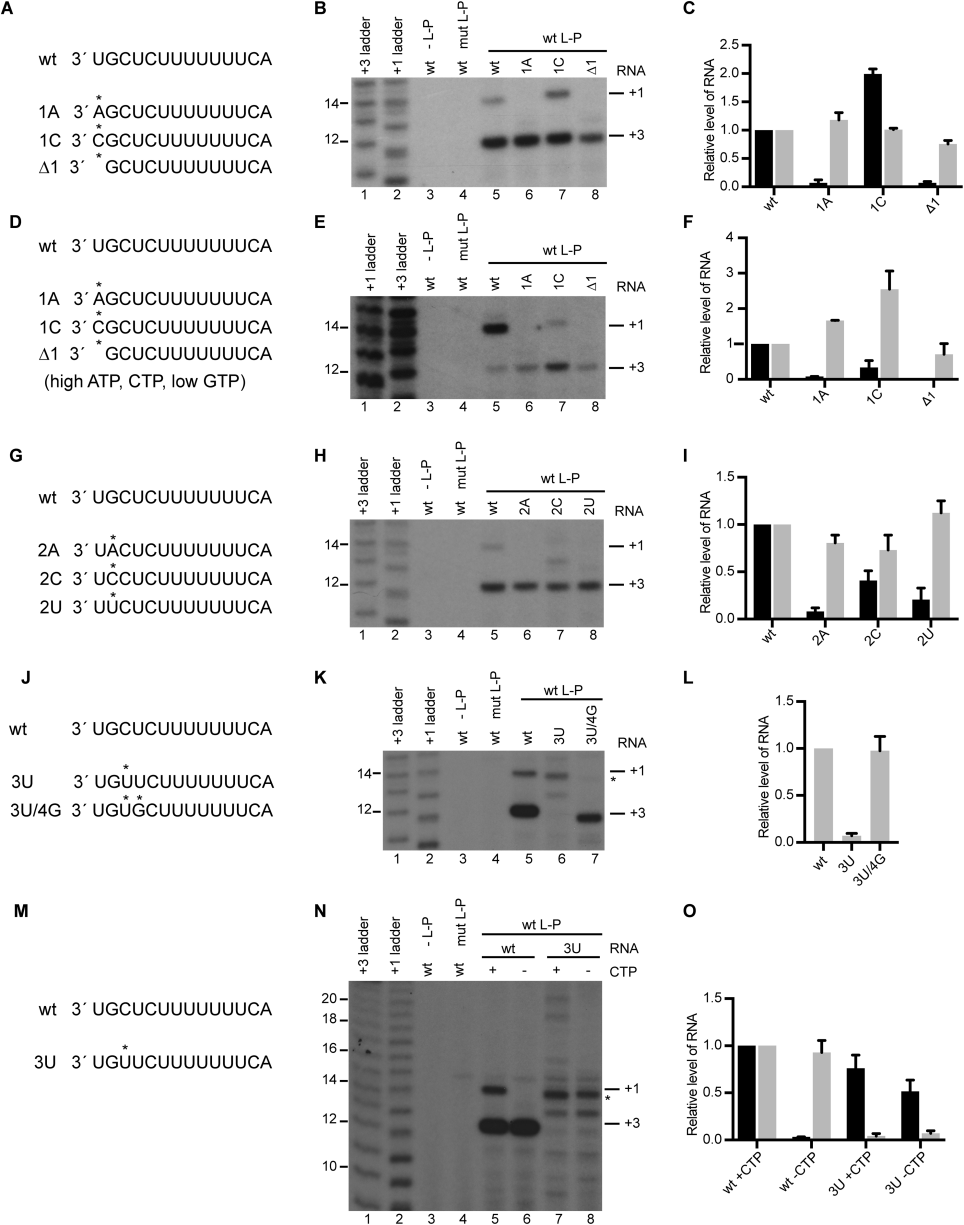
The RSV RdRp prefers to initiate opposite UG or C residues. (**A, D, G, J, M**) Schematic diagram showing the sequences of the wt and mutant templates. The asterisks indicate the positions of the mutations. (**B, E, H, K, N**) RNA synthesis products from wt *tr*14 template, or templates containing mutations at positions 1–3, as indicated. Lanes 1 and 2 contain ladders described in Figure 1, and lanes 3 and 4 are negative controls in which the L-P complex was omitted from the reaction, or contained a D811A substitution in the L protein, respectively. The experiments shown in panels B, H, K and N were performed with 1 mM ATP, CTP and GTP, and [α^32^P]-UTP tracer (with CTP omitted in some reactions in panel N); the experiment shown in panel E contained 1 mM ATP, 2 mM CTP, 10 μM GTP and UTP tracer. (**C, F, I, L, O**) Quantification of the 14 and 12 nt products (black and grey bars, respectively). Product levels were normalized to those in the corresponding reaction containing wt template. Panel L only shows 12 nt product because the 14 nt band generated from the mutant template was not initiated at +1. The data show the mean and standard error of three independent experiments, except for panels F and O, which show the mean and range of two independent experiments.

As noted in the Introduction, experiments in the minigenome system suggested that the RSV RdRp can select the initiating ATP or GTP independently of the template. Unfortunately, it was not possible to test this hypothesis directly, by examining binding interactions of L-P and NTPs in the absence of RNA, because the L protein contains additional GTP and ATP binding sites in the capping and methyltransferase domains, respectively, which would affect affinity measurements (30–32). Therefore, as an alternative means of testing the hypothesis, we examined how mutations at template positions 1–3 affected initiation. Analysis of T1 mutations showed that none significantly inhibited initiation from T3C, indicating that RdRp was still able to bind the template efficiently (Figure 4A–C). A T1A substitution or deletion (Δ1) inhibited RNA synthesis from +1, indicating that although the RdRp could bind the template, the identity of the nucleotide at the initiation site affected initiation efficiency. In contrast to the other mutations, a T1C substitution did not inhibit initiation from +1, but we consistently observed that the RNA produced migrated slightly differently than that from the wt template (Figure 4B, compare lanes 5 and 7). This would occur if the RNA product were of different sequence, suggesting that it might be initiated with GTP, rather than ATP. This possibility was tested by repeating the experiment using an NTP mix containing high concentrations of ATP and CTP and a low concentration of GTP (Figure 4D). Under these conditions, the intensity of the +1 band from the T1C mutant was low relative to that produced from the wt template, indicating that the RNA initiated from T1C was initiated with GTP, rather than ATP (Figure 4E, F). All three possible substitutions at T2 inhibited initiation from T1U, with little effect on initiation from T3C (Figure 4G–I). This showed that position T2G also plays an important role in enabling initiation from +1. Similar results were obtained with the *le* promoter (Supplementary Figure S1). Interestingly, a T2 G-to-C substitution in the *tr* promoter resulted in the appearance of a 13 nt band, indicating initiation from +2 (Figure 4H, lane 7).

A template containing a T3U substitution (Figure 4J, M) yielded complex results. This template did not generate a 12 nt band, and while a 14 nt band was detectable (indicated with an asterisk), unlike the other mutant templates tested, the T3U template yielded a ladder of bands (Figure 4K, lane 6; Figure 4N, lane 7). This raised the possibility that the substitution affected the ability of the RdRp to initiate precisely and augmented its tendency to stutter on the U tract in the promoter (4). If this were the case, the 14 nt band might not represent RNA initiated at T1U. To examine this possibility, experiments were performed using the wt and T3U templates, with CTP omitted from the reaction mix to inhibit initiation from +1. Whereas omission of CTP inhibited production of the 14 nt RNA from the wt template, most products from the T3U template were not affected (Figure 4N, O). This suggested that RNAs synthesized from the T3U template were initiated downstream of +2. Longer products were presumably due to excessive stuttering on the template. This finding indicates that position T3C plays an important role in stabilizing the initiation complex. Our previous study in the minigenome system had suggested that the RdRp can select ATP and CTP independently of the template to initiate at +1 (15). If this were the case, the presence of a UG motif at T3 and T4 might help anchor the RdRp and allow it to initiate at +3. To test this possibility a template containing a double substitution of 3U/4G was tested (Figure 4J). The additional substitution at T4 restored efficient initiation from +3 (Figure 4K, lane 7, L). These data show that T3C was important for anchoring the RdRp during initiation, but accurate initiation was restored if the template contained a UG motif at T3, T4.

Together, these results show that none of the substitutions at T1 and T2 inhibited the ability of the RdRp to bind the template as each resulted in initiation from T3C with equal or greater efficiency than from the wt template; in contrast T3C was an important element of the RdRp binding site. Although T1U and T2G did not appear to affect RdRp recruitment to the promoter, both these residues were required for the RdRp to initiate efficiently at +1. The data indicate that a C or UG motif at the initiation site in the template stabilized the initiation complex, with a C substitution at T1 or T2 allowing initiation from +1 or +2 with GTP, respectively, and a UG motif at T3, T4 allowing efficient initiation from +3. These results are consistent with a model in which the RdRp has an affinity for GTP or ATP and CTP, independently of the nucleotides at the initiation site of the promoter.

### The RdRp could initiate with a non-cognate NTP on a longer template

**Figure 5.**
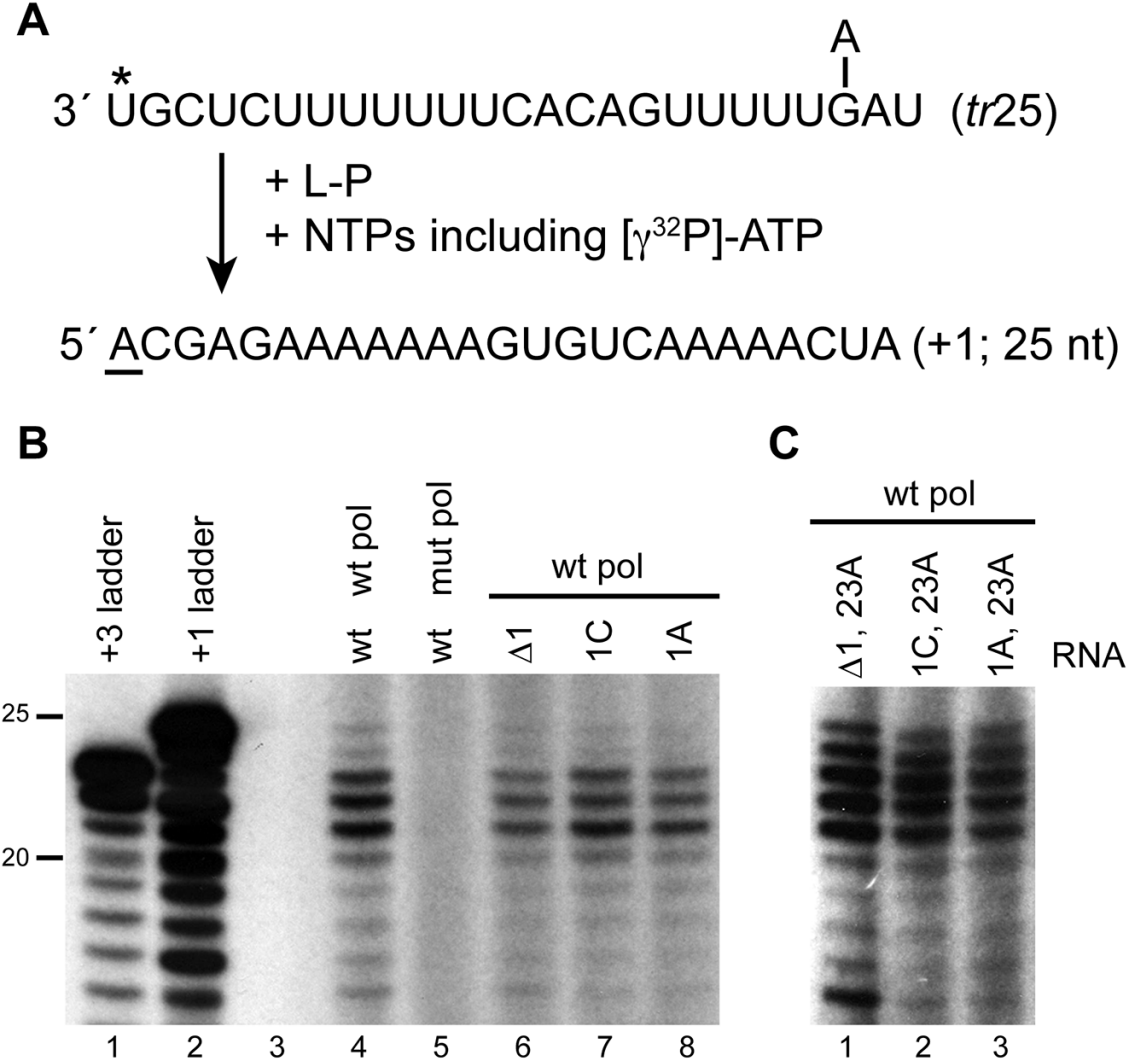
The RdRp could initiate with non-templated ATP on a *tr*25 template. (**A**) Schematic diagram illustrating how RNAs initiated with ATP were identified, as described in Figure 1C. Reactions contained a *tr*25 template with either a deletion or substitution at position 1 (indicated with an asterisk). Position 23, which was mutated from a G-to-A residue in some templates is indicated. (**B** and **C**) RNA products initiated with [γ^32^P]-ATP from the wt and mutant templates. RNA synthesis reactions contained 500 μM ATP, 1 mM CTP, 10 μM GTP and 500 μM UTP, and [γ^32^P]-ATP tracer. Panel B lanes 1 and 2 show the +3 and +1 ladders, prepared as described in Figure 1. Panel B, lane 5 is a negative control reaction performed with catalytically inactive D811A L protein, lane 3 is empty. The data in panel B are representative of five or more independent experiments (depending on the mutation); the data in panel C are representative of two independent experiments.

Although the conclusion that the RdRp can select initiating NTPs independently of template nucleotides is consistent with previous findings (15,26), the results obtained in Figure 4 differed in that in the minigenome system, deletion of T1U, or a T1U-to-C substitution resulted in replication product at 50–60% of wt levels, almost all of which was restored to wt sequence, whereas this was not the case in the experiments described above. As noted in the Introduction, this discrepancy could be due to a bias in the RNAs analyzed in the minigenome system. Alternatively, it could be because the conditions used *in vitro* did not support initiation with a sub-optimal complex in which the template and initiating NTPs did not match. During the course of this work, a crystal structure of the bunyavirus RdRp in complex with promoter RNA became available, and modeling based on this structure indicated that the RdRp could bind 20 nt of template RNA (33). We reasoned that if the RSV RdRp binds a similar template length, it was possible that the 14 nt template was too short to allow the RdRp, ATP and template to form a stable initiation complex without base-pairing between T1 and ATP. To test this hypothesis, we tested templates 25 nt in length and containing a deletion or substitution at T1U, and used [γ^32^P]-ATP as the trace nucleotide (Figure 5A). A similar pattern of products was generated from the wt and mutant templates, indicating that with a longer template, the RdRp could initiate with ATP irrespective of the identity or presence of position 1 (Figure 5B). We considered the possibility that an alternative explanation for the difference between the *tr*14 and *tr*25 templates was that in the case of *tr*25, the RdRp had the potential to enter the template internally at positions 22 and 23 and generate a pppApC primer that could subsequently be used to initiate at +1. Therefore, the experiment was repeated with templates containing a substitution at position 23. In each case the RdRp was able to initiate with ATP (Figure 5C). It should be noted that in these experiments the reactions contained high concentrations of ATP and CTP and a low concentration of GTP to enhance initiation at +1 and allow us to detect the products more readily. Nonetheless, this finding supports the conclusion that the RdRp can become preloaded with ATP and shows that initiation with a non-cognate NTP depends in part on RNA contacts downstream of nt 14 of the promoter.

### The RSV RdRp did not generate a pool of dinucleotide primer independently of the template

**Figure 6.**
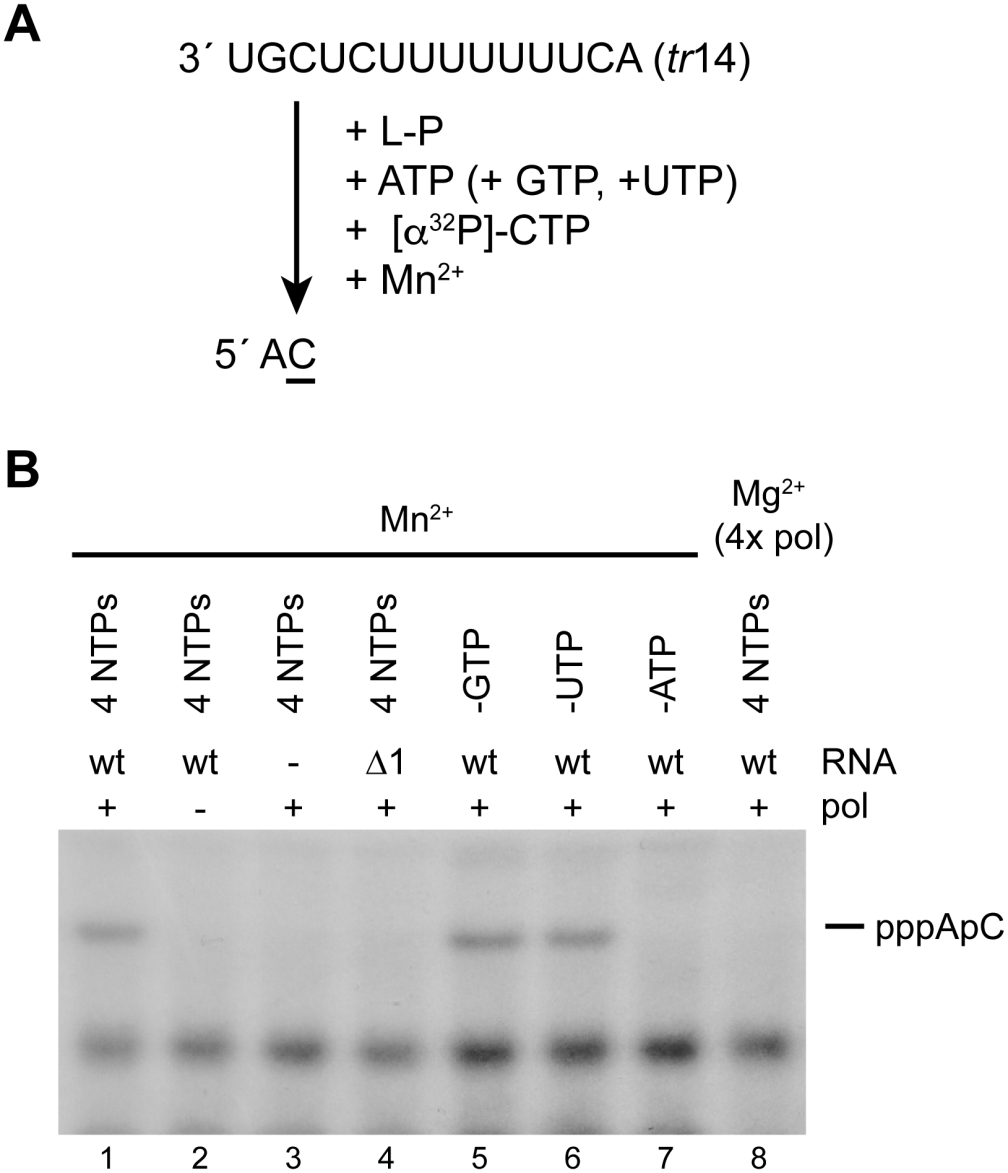
The RdRp could not efficiently synthesize a pppApC dinucleotide primer independently of the template. (**A**) Schematic diagram showing the requirements for production of a pppApC dinucleotide product. Reactions contained [α^32^P]-CTP and the site of radiolabel incorporation in the product is underlined. GTP and UTP are shown in parentheses because they were included in the positive control reaction, but could be omitted without affecting formation of the pppApC dinucleotide. (**B**) RNA products generated in the presence of all four NTPs (lanes 1–4 and lane 8) or in reactions from which either GTP, UTP or ATP was omitted (lanes 5–7). Reactions were performed either in the presence or absence of *tr*14 template RNA, or with *tr*14 RNA containing a deletion at position 1. Reactions were performed in the presence of Mn^2+^, rather than Mg^2+^, except that shown in lane 8. Only the region of the gel between the 1 and 2 nt markers is shown (note that the markers did not align with the bands because they contain a 5′ monophosphate, rather than triphosphate). This panel is one of five independent experiments.

The data presented in Figures 4 and 5 suggest that the RSV RdRp can select ATP and CTP, independently of the template to initiate at T1. Other RdRps have been shown to generate dinucleotide primers independently of the template (34,35). Therefore, we examined if the RSV RdRp was capable of polymerizing ATP and CTP in a template independent manner. In a control experiment containing a wt *tr*14 template and ATP, GTP, UTP and CTP, including [α-^32^P]-CTP a prominent two-nucleotide band could be detected provided the reactions contained Mn^2+^, rather than Mg^2+^, to facilitate initiation (Figure 6A and B, lanes 1 and 8) (4). The band could be detected if GTP or UTP were omitted from the reaction, but not if ATP was omitted (Figure 6B, lanes 5–7) indicating that it represented a pppApC dinucleotide. Reactions performed with all four NTPs, but in which the template was omitted did not yield the pppApC dinucleotide. This was also the case if the reaction contained a Δ1 *tr*14 template (Figure 6B, lanes 3 and 4). These results show that the RdRp does not have the capability to reiteratively produce AC dinucleotide in a template independent manner, although we could not rule out the possibility that it catalyzed phosphodiester bond formation but did not release the resulting dinucleotide, resulting in undetectable levels of dinucleotide product.

## DISCUSSION

The process of RNA synthesis initiation is complex, involving multiple players, including the RdRp, promoter and first two NTPs of the RNA product, as well as metal cations for catalysis (36). Each of these factors must be positioned appropriately, relative to the others, for initiation to be successful. This is even more challenging in the case of viruses that initiate RNA synthesis from the 3′ end of a linear template, as there is no template upstream of the initiation site to stabilize RdRp contacts. There is another layer of complexity in the case of RSV, in which the RdRp initiates RNA synthesis precisely at two closely spaced initiation sites within the genomic and antigenomic promoters. This unusual feature of RSV RNA synthesis, and the fact that initiation at both the +1 and +3 sites is required to allow the virus to perform replication and transcription, respectively, indicates that RSV must have evolved a specific initiation mechanism. The information presented here provides insight into the relationship between the two different initiation events, and the RdRp, NTP and template requirements for them to occur, and suggests a model for this initiation process.

Studies of vesicular stomatitis virus, another nsNSV in a different family, have indicated that there are two functionally distinct pools of RdRp, distinguished by associated proteins, and that one pool initiates at T1 of the *le*, to begin replication, and the other at the *gs* signal for the first gene, to begin transcription (37–39). In contrast, the data presented here clearly shows that RSV L and P alone are capable of replication and transcription initiation at the T1U and T3C sites (Figure 1). Another possible explanation for why the L-P complex could initiate at two different sites was that there could be functionally distinct sub-populations of RdRp, with one capable of initiating at T1U and the other at T3C. However, this is also not the case as varying the ATP or GTP concentration in the reaction resulted in an increase or decrease in initiation at T1U and T3C, with an inverse relationship between initiation at the two sites (Figures 2 and 3). This finding indicates that the L-P complex was undifferentiated before beginning RNA synthesis and that it became committed to initiation at T1U or T3C, depending on whether it associated with ATP or GTP.

The data indicate that RdRp becomes loaded with the initiating NTPs prior to binding the template, or at least, without reference to template nucleotides. Findings made with the minigenome and a 25 nt template containing a mutation of T1U indicated that the RdRp associates with ATP independently of this uracil (15,26) (Figure 5). Furthermore, the data indicate that not only is the RdRp capable of selecting ATP independently of T1U, but that it can become loaded with ATP and CTP, or GTP. Analysis of mutant *tr*14 templates showed that the RdRp could initiate at T1, T2, or T3 provided it was initiating opposite either a UG motif, or a C residue. The simplest explanation for the reason why the RdRp was stabilized opposite a UG or C motif is that in its pre-initiation form it has an innate affinity for ATP and CTP, or GTP. Other viral RdRps have also been shown to have innate affinity for some NTPs to begin initiation. While in some cases the bound NTP binds outside the catalytic site and has a structural role, in other cases the NTP is incorporated into polymerized product, as we propose occurs for RSV (34,35,40–42).

As noted above, ATP and GTP had opposing effects on the relative levels of initiation from T1U and T3C. In contrast, while CTP was essential for initiation at T1U, varying CTP concentration had no effect on initiation at T3C (Figure 2). These results indicate that ATP and GTP compete for the same binding site on the RdRp, whereas CTP binds another site; they also indicate that CTP does not influence ATP binding. Examination of the concentrations of ATP and GTP required for optimal initiation at T1U and T3C, respectively, showed that a lower concentration of GTP was sufficient for optimal initiation from +3, than the concentration of ATP required for optimal initiation from +1. This suggests that the polymerization domain of the RdRp has a greater affinity for GTP than ATP. If this were the case, this would help promote initiation at T3C. Furthermore, CTP was required at a very high concentration for optimal initiation at T1U, indicating that recruitment of CTP represents a barrier to initiation at this site (Figure 2E). This indicates that the initiating form of RdRp has only a low affinity for CTP. Given that CTP concentrations in cells would be expected to be approximately five-fold lower than those used in the assays described here, this would limit the level of replication initiation from T1U compared to transcription initiation from T3C. This would aid transcription at the expense of genome replication, and is likely to be important for efficient viral replication.

**Figure 7.**
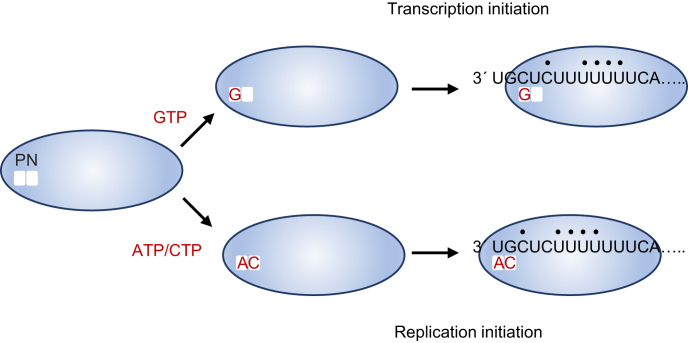
Model for transcription and replication initiation by the RSV RdRp. Schematic diagram illustrating different steps in initiation. The polymerization domain of the L protein is shown as a blue oval, with the P and N sites indicated. The RdRp can become loaded with GTP or ATP and CTP. The RdRp interacts with the promoter, possibly forming major contacts with the nucleotides indicated with a black dot. This would allow the RdRp to be positioned in two registers with respect to the template, with the P and N sites positioned opposite either T3 and T4, or T1 and T2, with the register being stabilized by Watson-Crick base-pairing between preloaded GTP or ATP/CTP, respectively. This would allow transcription initiation opposite T3C, or replication initiation opposite T1U. The model shows the *tr* promoter sequence that was utilized for most of this study. We propose that a similar sequence of events occurs during initiation from the *le* promoter.

In addition to NTP concentrations, the promoter sequence also played an important role in governing the relative levels of initiation from the two sites. We have previously shown that nucleotides 3, 5, 8, 9, 10 and 11 of the template are a core promoter element, important for both transcription and replication initiation (11). This element is almost identical to the *gs* signals at the beginning of each gene, and would be predicted to direct initiation from +3 (1,10). Consistent with this, initiation from +3 was dominant in almost every condition tested. This dominance was independent of relative affinity of the RdRp for GTP versus ATP because templates containing a C residue at T1 or T2 showed dominant initiation at T3C. Likewise, in the case of a 3U/4G template, in which the RdRp had the potential to initiate with ATP and CTP at T1 or T3, the dominant initiation site was at T3 (Figure 4). Together, these findings indicate that the RdRp preferentially binds the promoter such that its catalytic site is positioned opposite T3 and T4. The concept that the RdRp preferentially binds the promoter with its catalytic site positioned internally shares similarities with that of other RdRps. Seminal work on the mechanism of RdRp RNA synthesis initiation by analysis of crystal structures of bacteriophage phi6 RdRp, with or without template and NTPs, revealed that in the absence of NTPs, the RdRp bound the template such that the catalytic residues were aligned opposite T3 rather than T1. Upon binding NTPs, and their base-pairing with T1 and T2, the RdRp ratcheted back on the template to reposition T1 opposite the catalytic residues (43). Initial overshooting of the 3′ end of the template relative to the catalytic site has since been described for other RdRps, although it is not a universal feature (33,41,44–46). Inspection of the RSV promoter shows that the sequence at 5, 8, 9, 10 and 11 is also present at 3, 6, 7, 8 and 9, such that there is the potential for the RdRp to form similar interactions with template bases when its active site lies opposite T1 and T2 rather than T3 and T4 (Figure 7). Therefore, it seems likely that the RSV RdRp has stronger promoter interactions when located with its active site opposite T3 and T4, but has the potential to move between the two registers.

Together the data presented here and the information available for other viral RdRps suggest the following model for replication and transcription initiation in RSV (Figure 7). According to this model, the RSV polymerization domain contains at least two NTP binding sites, known as the priming (P) and incoming nucleotide (N) sites. Initially, the RSV RdRp exists as single undifferentiated pool consisting of a complex of the viral L and P proteins. The RdRp has the capability to bind ATP or GTP in its P site. ATP binding might affect the conformation of the N site, allowing CTP to bind specifically, or the N site might preferentially bind CTP. The RdRp binds the promoter, with a tendency to be positioned such that the P and N sites are opposite T3 and T4. If the RdRp is preloaded with GTP, it can recruit the appropriate NTP to base-pair with T4 and begin RNA synthesis directly from T3C to start the process of transcription. If the RdRp is pre-loaded with ATP and CTP, base-pairing between these NTPs and T1U and T2G alter the optimal template binding site, such that the RdRp is positioned with its P and N sites opposite T1 and T2. Having become positioned with the ATP and CTP opposite T1U and T2G, the RdRp could catalyze phosphodiester bond formation, and initiate RNA replication from +1.

If this model is correct, it indicates that the RSV RdRp has evolved to combine features present in other RdRps, such as specific interaction with initiating NTPs and a propensity for internal binding and template repositioning, to specifically and precisely initiate at two different sites within the promoter. In addition, it can explain how the virus has evolved to control relative levels of its different RNAs. During infection, RSV produces mRNA at higher levels than replicative RNAs, and genome RNA at a higher level than antigenome. The *le* and *tr* promoters differ at T4, with the *le* having a G rather than U residue at this site. If the N site has affinity for CTP, the difference in the nucleotide at T4 between the *le* and *tr* promoters could strongly promote transcription versus replication initiation at the *le* promoter, while allowing more efficient genome than antigenome initiation. Thus, together these findings indicate that RSV has evolved a simple and efficient mechanism to allow the RdRp to begin both mRNA transcription and genome replication from a single promoter, and synthesize appropriate levels of mRNA, antigenome and genome during infection.

## Supplementary Material

Supplementary Data
