# Supplementary material for: Mechanism for de novo initiation at two sites in the respiratory syncytial virus promoter
Source: Nucleic Acids Res. 2018 Jun 5;46(13):6785–96. doi: 10.1093/nar/gky480 (PMC6061868; doi:10.1093/nar/gky480)
Supplement: Supplementary Data [file gky480_supplemental_files.pdf]

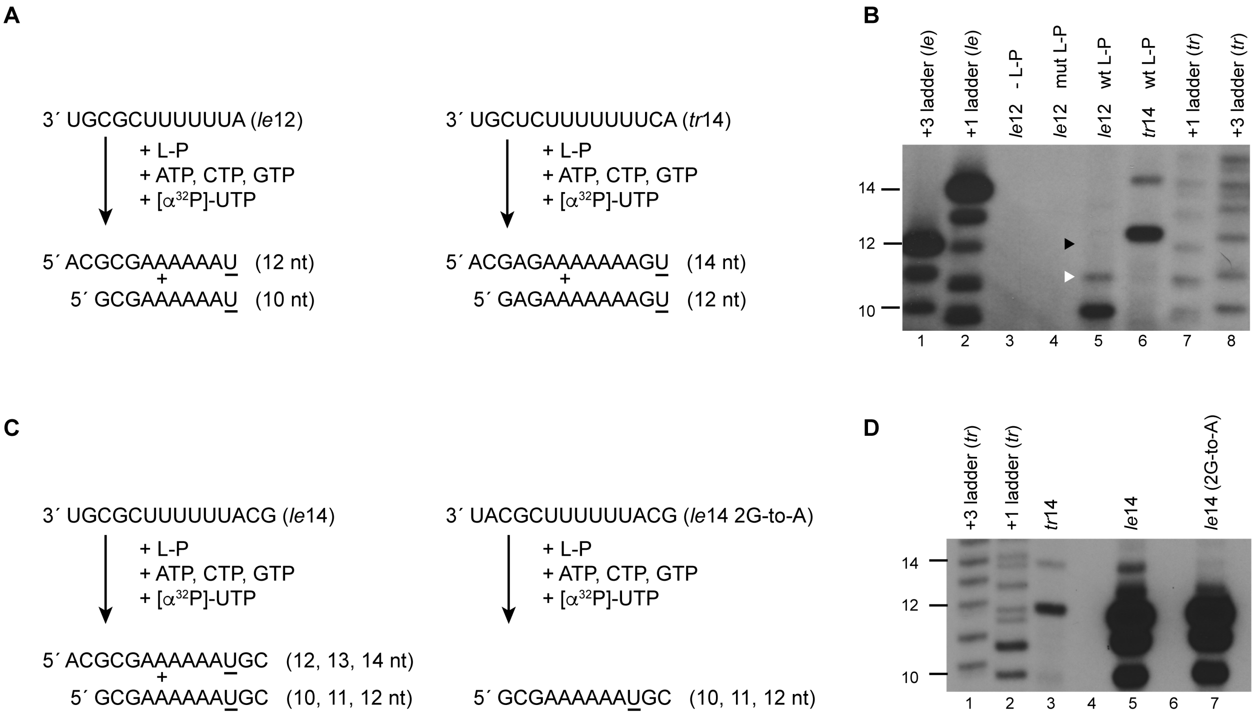

Figure S1. *In vitro* analysis of initiation sites in the *le* promoter. (A) Schematic diagrams showing the sequences of 12 nt *le* (*le*12) and 14 nt *tr* (*tr*14) promoter templates and the sequences and lengths of the radiolabeled products that would be generated from the T1U and T3C sites. In this experiment, a 12 nt *le* promoter template was used because the first A residue is at position 12 of the template. (B) RNA products generated in the presence of [α^{32}P]-UTP. Reactions contained 1 mM each of ATP, CTP and GTP, and [α^{32}P]-UTP tracer. Lanes 1, 2, 7 and 8 show ladders consisting of [γ-^{32}P] labeled RNA which had been subjected to alkali digest. Lanes 1 and 2 utilized 12 and 14 nt labeled RNAs corresponding in sequence to RNA initiated at the +3 or +1 sites, respectively, of the *le* promoter; lanes 7 and 8 utilized 25 or 23 nt labeled RNA corresponding in sequence to RNA initiated at the +1 or +3 sites, respectively, of the *tr* promoter. Lanes 3 and 4 are negative control reactions in which the L-P complex was omitted from the reaction, or substituted with L-P containing catalytically inactive D811A L protein. The black arrowhead shows the position of the RNA produced from T1U of the *le*12 promoter. It can be seen that this is produced at a significantly lower level than the RNA initiated at T1U of the *tr*14 promoter, relative to initiation from +3. The identity of the 11 nt band indicated with the white arrowhead is not known; it might represent RNA initiated at T3C that had been elongated one additional nucleotide due to RdRp stuttering on the U tract within the promoter. (C) Schematic diagrams showing the sequences of the wt *le*14 template and a *le*14 template containing a substitution at position 2G (*le*14 2G-to-A), which was expected to inhibit initiation from T1U promoter (see main results text), and the sequences and lengths of the radiolabeled products that would be generated from the T1U and T3C sites. Note that in this case, multiple bands are expected from each initiation reaction because the [α^{32}P]-UTP incorporation site lies three nucleotides before the end of the template. (D) RNA products generated in the presence of [α^{32}P]-UTP. Reactions contained 1 mM each of ATP, CTP and GTP, and [α^{32}P]-UTP tracer. Lanes 1 and 2 show ladders corresponding to RNA initiated at the +3 or +1 sites, respectively, of the *tr* promoter. Lanes 4 and 6 were empty. This analysis shows that the *le* promoter also signaled RNA

synthesis from T1U and T3C, but with a much greater bias towards T3C initiation than the *tr* promoter. The data are representative of three independent experiments.

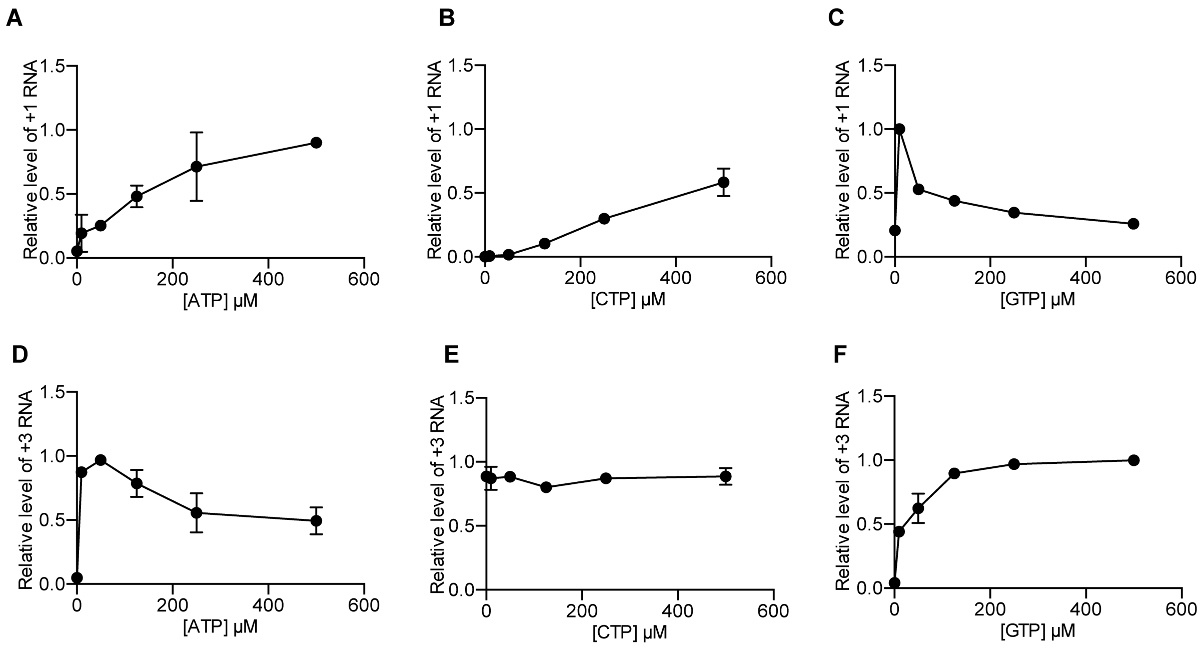

Figure S2. Effect of NTP concentrations on initiation at T1U or T3C. Quantification of the products from the +1 (A-C) and +3 (D-F) initiation sites under conditions of varying NTP concentration. The graphs show the same data as presented in Figure 2, but focusing on the 0-500 µM NTP range to show the effects of low NTP concentrations in more detail.
